# Unusual case of left ventricular ballooning involving the inferior wall: a case report

**DOI:** 10.1186/1757-1626-2-140

**Published:** 2009-02-20

**Authors:** Andrea Rognoni, Marzia Bertolazzi, Sergio Maccio', Danilo Reale, Riccardo Proietti, Giorgio Rognoni

**Affiliations:** 1 Division of Cardiology, Ospedale Sant' Andrea, Vercelli, Italy

## Abstract

**Background:**

Tako – tsubo like syndrome (also named left ventricular apical ballooning) is an unusual cardiomyopathy with an high incidence in Japanese population of female sex, following an emotional stress. The clinical features (typical chest pain), and the electrocardiographic changes (negative T wave and persistent ST elevation in anterior leads), are suggestive of an acute myocardial infarction; nevertheless the coronary angiography show coronary arteries without lesions and the ventriculography show specific segmental dysfunction. In the literature there are many reports of typical left ventricular ballooning (apical); due to the rarity of the atypical localizations (such as mid, basal, anterior or inferior left ventricular wall) many authors think they are different physiopatologic entity.

**Case report:**

We report a case of 50 – years old woman, with a family history of ischeamic cardiomyopathy but with no additional cardiovascular risk factors, who arrived to emergency department with a recent episode of chest pain (about 30 minutes) with electrocardiographic and echocardiographic features suggested of a inferior ST elevation myocardial infarction. Coronary angiography showed coronary arteries without atherosclerotic lesions; ventriculography showed an inferior dysfunction.

**Conclusion:**

This data can suggest for an atypical form (in term of clinical presentation and localization) of left ventricular ballooning involving the inferior wall (never described in the literature), not preceded by any emotional or physical stress. The follow – up performed by transthoracic echocardiography (2 months later) revealed a complete regression of wall motions abnormalities.

## Background

The left ventricular "apical ballooning" syndrome, also known as "Tako – Tsubo like syndrome", has recently been the subject of numerous studies and reports in the literature, to the point that the term "stress-induced cardiomyopathy" [1] has been coined. It was initially described by Japanese authors in the early Nineties (hence the name Tako – tsubo, because of the characteristic shape assumed by the left ventricle in telesystole, wholly similar to that of octopus traps in Japan) and its clinical presentation in most cases mimics acute myocardial infarction; in the past five years, several European, American and Australian cases have also been reported in the literature [2,3].

The incidence of the pathology is estimated to be approximately around 1% – 2% among all patients who come under cardiological attention for acute ischemic events (both acute coronary syndrome and acute myocardial infarction); according to a recent statistic by the American Heart Association, out of 732,000 yearly dismissals of patients with a primary diagnosis of acute myocardial infarction, a number varying between 7,000 and 14,000 patients may present stress-induced cardiomyopathy [4]. However, an accurate estimate of incidence is not feasible because of its recent definition, disparate clinical presentations and constant evolution.

The physiopathological motive seems to be linked to the plasmatic release of catecholamines as a result of intense emotional and/or physical stress with their consequent direct damage in terms of the metabolism of cardiac myocytes; high levels of catecholamines reduce myocyte activity through an overload of calcium mediated by cyclic adenosinmonophosphate; catecholamines are also sources of free radicals derived from oxygen and, in animal models, by interfering with the transmembrane transport of sodium and calcium, they cause a myocyte dysfunction, increasing the concentration of intracellular calcium [5,6].

In 2006 the American Heart Association included "stress-induced cardiomyopathy" in the classification of cardiomyopathies as a "primitive and acquired cardiomyopathy" which can involve the entire extension of the left ventricle and no longer just the apex, as described initially[2]; however, in the literature there are limited reports of left ventricular ballooning with atypical localization at medium ventricular level, at the base of the ventricle, at the level of the lower wall and of the front wall.

We describe a case of ventricular ballooning localized at the level of the inferior wall in a patient who came to our observation in the absence of emotional and physical stresses identifiable by the anamnesis, chest pain and electrocardiographic alterations characteristics for acute ST elevation myocardial infarction (STEMI).

## Case report

**Figure 1:**
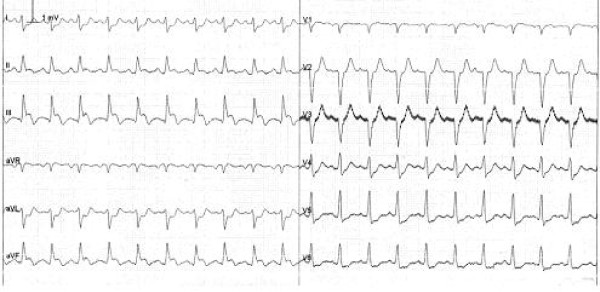
**electrocardiogram at the admission**.

A 50-year old woman, known to have surgically treated congenital hydrocephalous, with a family history of ischemic cardiopathy but with no additional cardiovascular risk factors, without a recent major psychological or physical stresses, was admitted to our Emergency Department for prolonged chest pain felt while at rest, continuos and not irradiated, lasting for about 45 minutes and partially reduced spontaneously upon arrival at the hospital. The physical examination did not yield any pathological findings; blood pressure was 100/70 mmHg. Entry electrocardiogram showed the presence of sinusal rhythm and ST elevation in the inferior leads and light ST depression in lateral (Figure 1).

The echocardiogram performed at the time of admission indicated only a hypokinesia of the infero-basal wall with normal systolic function (ejection fraction 54%) and absence of valve pathologies of relevance. The first hematochemical exams showed a significant increase in cardio-specific enzymes (troponin I 3.12 ng/ml, Ck – Mb 11,1 ng/ml, BNP 98.7 pg/ml). After the start of intravenous medical therapy with nitrates, sodic heparin (5000 U in bolus and 1000 U/hour), inhibitors of the glycoprotein IIB/IIIa (abciximab) and orally with beta blocker (atenolol 100 mg), atorvastatin (40 mg) and clopidogrel (300 mg) the patient was subjected to a urgent coronary angiography.

**Figure 2:**
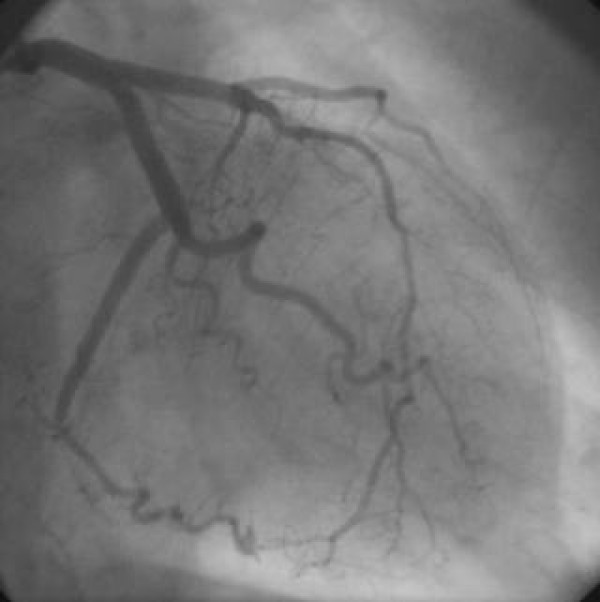
**normal left coronary artery**.

**Figure 3:**
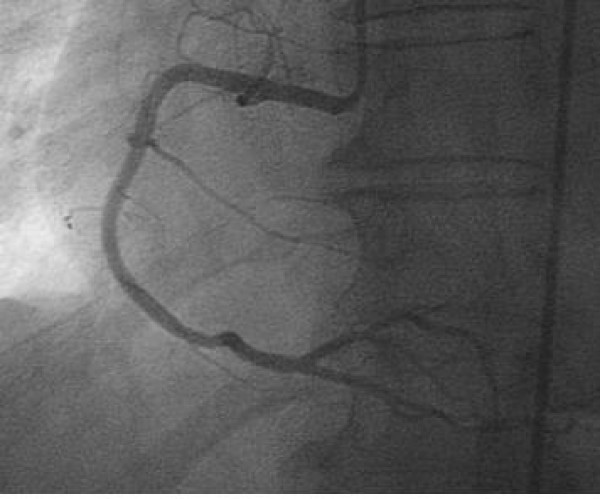
**normal right coronary artery**.

**Figure 4:**
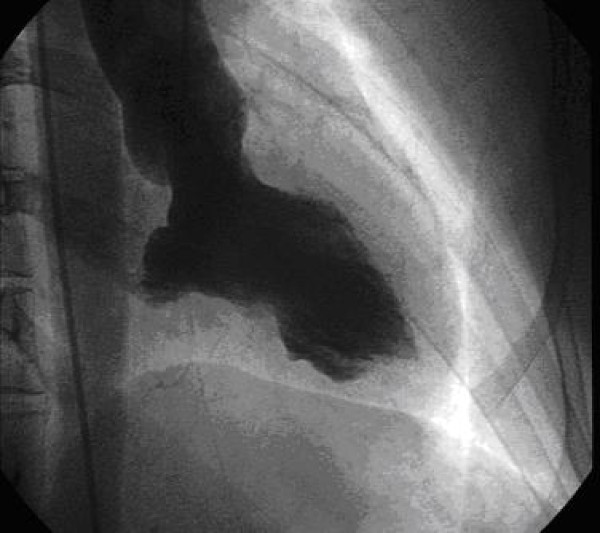
**inferior ventricular ballooning (telesystole of the left ventricle)**.

The procedure showed epicardic coronaries free of lesions (Figure 2, Figure 3); however, the left ventriculography highlights the presence of a dyskinesia limited to the lower wall (Figure 4). During the hospital stay, an evaluation of the plasmatic and urinary catecholamines was performed, which showed an increased presence of vanilmandelic acid (21 ng/24 hour); serum concentration of brain natriuretic peptide was 60.5 pg/ml (normal range 0.0 – 18.0 pg/ml), epinephrine was 567 pg/ml (normal range 25 – 50 pg/ml), norepinephrine was 890 pg/ml (normal range 150 – 350 pg/ml). The patient was discharged home on the fifth day and a follow – up performed by transthoracic echocardiography (2 months later) revealed a complete regression of wall motions abnormalities

## Discussion

In the literature, in recent years, there have been numerous reports of patients with transitory dysfunction of the left ventricle in relation to physical stress or to episodes of important psychic stress [7,8]. The first descriptions dating back to the early Nineties by Japanese authors allowed to identify this pathology with the first name of "tako – tsubo like syndrome" because of the characteristic shape assumed by the left ventricle in telesystole and wholly similar to the shapes of the respective Japanese octopus trap [9].

As early as 2002, Karisu et al[10] highlighted the clinical and instrumental characteristics of this pathology: aged female patients (sixth and seventh decade of life); symptoms that specifically mimic an ischemic heart attack with benign evolution; electrocardiographic alterations with T wave inversion and occasionally persistence of the over-leveling of the ST segment in the absence of obstructive coronary pathology; echocardiographic alterations suggesting a multicoronary involvement with resolution in a few weeks. Based on these data and on those obtained in their own center, in 2004 Bybee et al[11] proposed the "Mayo Clinic criteria for apical ballooning syndrome".

However, etiology does not seem altogether clear; Karisu et al demonstrated, through the histological analysis of the myocardial muscle tissue, performed through endomyocardial biopsy, the presence of focal areas of myocytolysis and of infiltration by mononucleated cells and free connective tissue [10]; moreover, biochemical analyses showed negativity for Adenovirus, Coxsakievirus, Cytomegalovirus and Echovirus. Ueyama et al. obtained an animal model of ventricular ballooning in laboratory guinea pigs; these animals, after a stressful event characterized by immobilization, showed an increased secretion of "heat shock protein 70", of "brain natriuretic peptide" in addition to an increased activation of proto – oncogenic – fos and of – jun [11,12].

The most creditable etiology and physiopathology hypothesis seems to be that as a result of mental and/or physical stress there is an increase in the plasmatic release of catecholamines (as is evident also in our own two cases) and that said plasmatic concentration is able to determine a direct effect on myocyte metabolism. High levels of catecholamines reduce myocyte activity [13] through an overload of calcium mediated by cyclic adenosinmonophosphate; catecholamines are also sources of free radicals derived from oxygen and, in animal models, by interfering with the transmembrane transport of sodium and calcium, they cause a myocyte dysfunction, increasing the concentration of intracellular calcium. Bolli and Marban have shown that concentrations of catecholamines are associated, from the tissue viewpoint, to the presence of necrosis of muscular contraction bands, of eosinophil infiltrates and of interstitial inflammation with hyperexpression of mononucleated cells [14,15].

However, our case completely deviate from the characteristics just described, both in the anamnestic absence of evident psycho-physical stress and in the localization of the ventricular ballooning (inferior wall).

The typical apical localization of the ballooning is not quite clear: it seems to be linked to a greater response to the adrenergic stimulus at the level of the apex of the myocardium (the norepinephrine content at the apex is lower than at the base); moreover, at the human heart level there is a heterogeneous distribution of nervous terminations. The ventricular apex may be no more vulnerable to the adrenergic stimulus than the medium – ventricular region and the base; this physiological variation of the regional vulnerability of the left ventricle could be responsible for the different localizations.

An additional alternative, recently proposed [16], which can explain the different localizations of the ventricular kinetics deficits, is linked to the presence of a perfusion gradient between the base, the medial segments and the apex of the ventricle; said gradient has been demonstrated both in patients with multiple cardiovascular risk factors and coronary pathology and in patients with no evidence of obstructive coronary disease; however, it has not been demonstrated to be present in patients without risk factors. In the literature, in recent years, some cases of ballooning with atypical localization have been reported; the apical localization of the kinetic alterations is no longer considered an indispensable element for diagnostic and clinic determinations. It is likely that atypical localizations are generically included among "acute coronary syndromes with normal coronaries" and for this reason their actual incidence is underestimated [[17], 18, 19].

The current common international scientific opinion agrees that the "ventricular ballooning syndrome" has the same origin regardless of localization, therefore we can speak of "all sides of the same coin". However, there are some differences between the clinical characteristics of patients with non apical ballooning and those with apical ballooning: the former present more intense heart failure and absence of electrocardiographic alteration. The peculiarity of our case regards, in addition to the atypical and rare inferior localization, above all the absolutely abnormal manifestation (STEMI), with no psycho – physical stress.

## Conclusion

In conclusion we described and explained a rare case of atypical left ventricular ballooning involving the inferior wall presented to our attention mimicking an acute inferior myocardial infarction.

## Consent

Written informed consent was obtained from the patient for publication of this case report and accompanying images. A copy of the written consent is available for review by the Editor – in Chief of this journal.

## Competing interests

The authors declare that they have no competing interests.

## Authors' contributions

All authors have made substantial contribution to concept this case report.

## References

[B1] Prasad A Lerman A Rihal CS Apical ballooning syndrome (Tako – Tsubo or stress cardiomyopathy): A mimic of acute myocardial infarction Am Heart J 2008 155 408 17 10.1016/j.ahj.2007.11.008 18294473

[B2] Maron BJ Towbin JA Thiene G Antzelevich C Corrado D Arnett D Moss AJ Seidman CE Young JB American Heart Association contemporary definitions and classification of the cardiomyopathies: American Heart Association scientific statement from the Council on clinical Cardiology, Heart Failure and Transplantation Committee; Quality of Care and Outcomes Research and Functional Genomics and Translational Biology Interdisciplinary Working Groups; and Council on epidemiology and Prevention Circulation 2006 113 1807 16 10.1161/CIRCULATIONAHA.106.174287 16567565

[B3] Abdulla I Kay S Mussap C Nelson Gl Rasmussen HH Hansen PS Ward MR Apical sparing in Tako – Tsubo cardiomyopathy Int Med J 2006 36 414 8 10.1111/j.1445-5994.2006.01095.x 16780446

[B4] Bybee KA Prasad A Barsness G Lerman A Jaffe AS Murphy JG Wright RS Rihal CS Clinical Characteristics, outcomes, and impaired myocardial microcirculation in patients with transient left ventricular apical ballooning syndrome: a case series from a U.S medical center Am J Cardiol 2004 94 343 6 10.1016/j.amjcard.2004.04.030 15276100

[B5] Mann DL Kent RL Parson B Cooper G Adrenergic effects on the biology of the adult mammalian cardiocyte Circulation 1992 85 790 804 1370925 10.1161/01.cir.85.2.790

[B6] Singal PK Kapur N Dhillon KS Beamish RE Dhalla NS Role of free radicals in catecholamine – indeiced cardiomyopathy Can J physiol Pharmacol 1982 60 1390 7 7151008 10.1139/y82-207

[B7] Tsuchihashi K Ueshima K Uchida T Oh-mura N Kimura K Owa M Yoshiyama M Miyazaki S Haze K Ogawa H Honda T Hase M Kai R Morii l Transient left ventricular apical ballooning without coronary artery stenosis: A novel heart syndrome mimicking acute myocardial infarction J Am Coll Cardiol 2001 38 11 18 10.1016/S0735-1097(01)01316-X 11451258

[B8] Wittstein IS Thiemann DR Lima JA Gaughman KL Schulman SP Gerstenblith G Wu KC Rade JJ Bivalacqua TJ Champio HC Neurohumoral features of myocardial stunning due to sudden emotional stress N Engl J Med 2005 352 539 548 10.1056/NEJMoa043046 15703419

[B9] Ishihara M Sato H Tatehishi "Tako – Tsubo" type cardiomyopathy Kokyo to Junkan 1997 45 879 885

[B10] Kurisu S Sato H Kawagoe T Ishihara M Shimatani Y Nishioka K Kono Y Umemura T Nakamura S Tako – Tsubo – left ventricular dysfunction with ST – segment elevation: a novel cardiac syndrome mimicking acute myocardial infarction Am Heart J 2002 143 448 55 10.1067/mhj.2002.120403 11868050

[B11] Bybee KA Kara T Prasad A Lerman A Barsness GW Wright RS Rihal CS Transient left ventricular apical ballooning syndrome: a mimic of St – elevation myocardial infarction Ann Inter Med 2004 141 858 65 10.7326/0003-4819-141-11-200412070-00010 15583228

[B12] Ueyama T Sanba E Kasamtsu K Molecular mechanism of emotional stress – induced and cathecolamine – induced heart attack J Cardiovasc Pharmacol 2003 41 Suppl 1 S115 S118 12688407

[B13] Grouzmann E Fathi M Gillet M de Torrentè A Cavadas C Brunner H Buclin T Disappearance rate of cathecolamines, total metanephrines, and neuropeptide Y from plasma of patients after resection of pheocromocytoma Clin Chem 2001 47 1075 82 11375294

[B14] Bolli R Marban E Molecular and cellular mechanisms of myocardial stunning Physiol Rev 1999 79 609 34 10221990 10.1152/physrev.1999.79.2.609

[B15] Yong-Hahn J Gwon HC Woo-Park S The clinical features of transient left ventricular nonapical ballooning syndrome: Comparison with apical ballooning syndrome Am Heart J 2007 154 1166 73 10.1016/j.ahj.2007.08.003 18035091

[B16] Hernandez-Pampaloni M Keng FY Kudo T Abnormal longitudinal base – to – apex myocardial perfusion gradient by quantitative blood flow measurements in patients with coronary risk factors Circulation 2001 104 527 32 10.1161/hc3001.093503 11479248

[B17] Mazzarotto P Stecconi P Gemelli F Azzarito M Farnetti F Un caso di "ballooning sindrome" con localizzazione atipica anteriore Ital Heart J 2005 6 11 730 734 16318248

